# Mapping quantitative trait loci for kernel composition in almond

**DOI:** 10.1186/1471-2156-13-47

**Published:** 2012-06-21

**Authors:** Rafel Socias i Company

**Affiliations:** 1 Unidad de Fruticultura, Centro de Investigación y Tecnología Agroalimentaria de Aragón (CITA), Av. Montañana 930, 50059 Zaragoza, Spain; 2 Present address: Unidad de Pomología, Estación Experimental de Aula Dei (EEAD-CSIC), PO Box 13034, 50080 Zaragoza, Spain; 3 Present address: Laboratorio de Mejora Genética y Biología Molecular, Parque Científico Tecnológico de Aula Dei (PCTAD), Av. Montañana 930, 50059 Zaragoza, Spain

**Keywords:** Almond, *Prunus amygdalus* Batsch, Kernel composition, Quality, SSRs, QTL, Genetic map, Breeding

## Abstract

**Background:**

Almond breeding is increasingly taking into account kernel quality as a breeding objective. Information on the parameters to be considered in evaluating almond quality, such as protein and oil content, as well as oleic acid and tocopherol concentration, has been recently compiled. The genetic control of these traits has not yet been studied in almond, although this information would improve the efficiency of almond breeding programs.

**Results:**

A map with 56 simple sequence repeat or microsatellite (SSR) markers was constructed for an almond population showing a wide range of variability for the chemical components of the almond kernel. A total of 12 putative quantitative trait loci (QTL) controlling these chemical traits have been detected in this analysis, corresponding to seven genomic regions of the eight almond linkage groups (LG). Some QTL were clustered in the same region or shared the same molecular markers, according to the correlations already found between the chemical traits. The logarithm of the odds (LOD) values for any given trait ranged from 2.12 to 4.87, explaining from 11.0 to 33.1 % of the phenotypic variance of the trait.

**Conclusions:**

The results produced in the study offer the opportunity to include the new genetic information in almond breeding programs. Increases in the positive traits of kernel quality may be looked for simultaneously whenever they are genetically independent, even if they are negatively correlated. We have provided the first genetic framework for the chemical components of the almond kernel, with twelve QTL in agreement with the large number of genes controlling their metabolism.

## Background

Almond (*Prunus amygdalus* Batsch) is a major tree nut grown in areas of Mediterranean climate. The kernel is the edible part of the nut and is considered an important food with a high nutritional value. It may be consumed raw or cooked, blanched or unblanched, combined and/or mixed with other nuts. It can also be transformed to be incorporated into other products or to produce marzipan and nougat [1]. Almond kernel quality was until recently defined exclusively by physical parameters: size, shape, double kernels, etc. However, the different uses of almond may require kernels with a specific composition, depending on each commodity.

The main fraction of the almond kernel is the lipid fraction, which confers a high nutritive value. This lipid content constitutes an important caloric source but does not contribute to cholesterol formation in humans, due to their high level of unsaturated fatty acids, mainly monounsaturated, which are negatively correlated with serum lipid profiles and cholesterol status associated with a lower risk of cardiovascular diseases [2,3]. The major fatty acids in the almond kernel are oleic (70-80 % of total fatty acid content), linoleic (10-17 %) and palmitic (5.5-6.5 %). This high oleic acid concentration makes almond a very important part of the human diet, as oleic acid is known to reduce low-density lipoprotein cholesterol without altering beneficial high lipoprotein cholesterol levels. Kernel tendency to rancidification during storage and transport is a quality loss and is related to oxidation of the kernel fatty acids [4]. Thus, oil stability and fatty acid composition are considered an important criterion to evaluate kernel quality [5].

Most vegetable oils, especially oils with high levels of unsaturated fatty acids, contain tocopherols in differing amounts. Tocopherols are natural mono-phenols occurring in plants as a family of four different homologues depending on the position and number of methyl groups. These components are believed to be involved in a diversity of physiological, biological and biochemical functions, mainly due to their role as antioxidants [6]. Their main biochemical function is believed to be the protection of poly-unsaturated fatty acids against peroxidation [7]. Alpha-tocopherol is the form of Vitamin E that is most efficiently used by the human body and is often deficient in modern diets [8,9]. Vitamin E, the antioxidant polyphenols and dietary fiber from almonds help prevent heart disease and cancer [7,10]. Thus, tocopherol content in seed oils is considered as a value-added compound [11], not only for the quality of the human diet, but also for maintaining the stability of almond quality [12,13].

Almonds are also a very good source of dietary protein, as approaching that of red meat [1], with an average of 20 g protein per 100 g, depending on cultivars. In addition, almond protein is efficiently digested, absorbed, and utilized [14]. All these nutritional and healthy aspects are receiving greater attention from the general public in recent years and have just been reviewed [15].

Until recently almond breeding has focused on selecting self-compatible and late-blooming cultivars with fruits of a high physical quality [16]. Consequently, very little information on chemical evaluation of the almond kernel has been found and the studies carried out to determine the chemical components of the almond kernel and their variability are scarce [13,17-19]. Incorporation of such analyses in the evaluation of new plant material would be of great interest in determining the possible commercial and industrial use of the product, since the specific use of the kernel depends primarily on its chemical composition [1]. Additionally, in recent years, food and health aspects are receiving special attention from the general public. The determination of food authenticity and origin is a crucial issue in food quality control and safety [20].

The chemical composition has hardly been considered as an objective in almond breeding programs [1,12,21]. Consequently, it has not been genetically approached and the heritability of the different kernel components has only been considered recently [22]. Conventional fruit breeding has traditionally been a slow process involving enormous resources of time, labor and land, including field management and observations of field trials [23]. Since most observations cannot be carried out until several years after planting the seedlings, the use of DNA markers would greatly increase the speed of the breeding process. The development of marker-assisted selection techniques would allow decisions to be made at the nursery stage in order to decide which individuals should be retained and which should be culled. Any tool helping to identify the different levels of expression of the kernel chemical components would be essential in an almond breeding program in order to select new genotypes with improved kernel quality.

SSR markers have become a very useful tool for constructing linkage maps and locating genes controlling phenotypic variability. More than 10 molecular genetic maps have been constructed for different *Prunus* species [24] Among these maps, that obtained from the cross ‘Texas’ almond × ‘Earlygold’ peach *P. persica* (L.) Batsch] (T × E), is considered the reference *Prunus* map [25]. A total of 827 markers covering a total distance of 524 cM have been placed on this map [26]. Additionally, the high level of synteny between the genome of the different *Prunus* species [27], has allowed the map positions of 28 major genes affecting agronomic traits and more than 20 QTL to be established [28]. However, no studies have been undertaken on mapping the chemical traits related to almond kernel quality.

The study of the almond cross ‘Vivot’ × ‘Blanquerna’ (V × B) has allowed a linkage map of this progeny to be constructed [29], and the chemical composition of the kernels of its plants to be determined [22]. Thus, our objective was to identify QTL associated with the different chemical components of the almond kernel and thus establish a genetic tool to be applied in an almond breeding program aiming at increased kernel quality.

## Results and discussion

### Linkage map of QTL controlling the chemical components of the almond kernel

**Figure 1:**
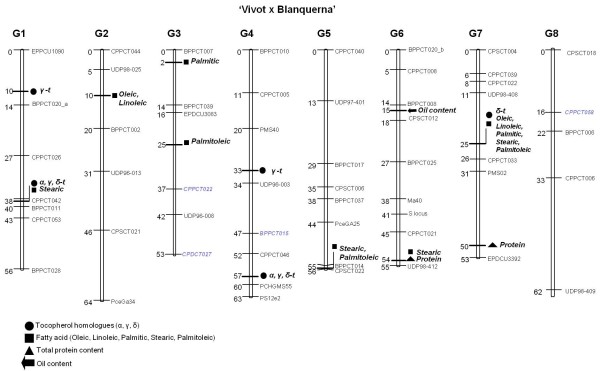
**Combined linkage map showing the location of QTLs for chemical composition of the almond kernel.** Combined linkage map of the ‘Vivot’ Í ‘Blanquerna’ population constructed using _MAPCHART V_. 2.1 [71] with the location of the QTLs related to the chemical composition of the almond kernel. The newly incorporated markers are in blue italics.

The population studied was selected because of the wide range of variability of chemical components of their kernels. This population belongs to the CITA almond breeding program and was obtained from the V × B cross. A map from this population has already been published [29] and has been used for detecting QTL controlling the chemical components of the almond kernels for the first time. This map, previously constructed with 52 SSR markers, has been increased with 4 more SSRs, representing a total of 56 markers (Table 1). The position of these markers (Figure 1) agrees with the last almond map published [30]. An LOD score of 2.0 was used to declare the presence of a QTL linked to all traits studied (total protein and oil contents, and the percentages of the main fatty acids and the main tocopherol homologues). A total of 12 putative QTL controlling these traits were detected in this analysis, corresponding to seven genomic regions of the eight almond LGs. Only the LG8 did not show any QTL for almond kernel composition. Some QTL were clustered in the same region or shared the same molecular markers (Table 2). The LOD values for any given trait ranged from 2.12 to 4.87, explaining from 11.0 to 33.1 % of the phenotypic variance of the trait.

**Table 1:** **SSRs used in the identification of QTL in the almond cross ‘Vivot’** × **‘Blanquerna’**

**Species of origin**	**SSR name**	**Reference**	**No of SSRs tested**	**No of SSRs amplified**	**No of SSRs mapped**	**No of loci mapped**	**Percentage of total SSRs placed in the ‘V × B’ map**
Peach	BPPCT	[31]	24	23	15	16	28
Peach	CPPCT	[32]	32	31	15	15	27
Japanese Plum	CPSCT	[33]	6	6	6	6	12
Almond	EPDCU	[34]	6	6	2	2	3
Peach	EPPCU	[34]	9	9	1	1	2
Peach	PCHGMS/Ma0	[35,36]	5	5	2	2	4
Peach	UDP	[37,38]	17	17	9	9	15
Cherry	Others	[39,40]	11	9	6	6	9
-	Total	-	110	106	56	57	100

**Table 2:** QTL for chemical traits in almond found to be significant with the linkage group (LG), LOD score, nearest marker and percentage of variance explained (% Exp)

	**Trait**	**LG**	**LOD**	**Nearest SSR marker**	**% Exp**
**Tocopherol homologues**	α-tocopherol	1	3.21	CPPCT042	20.0
		4	2.26	PCHGMS55	17.8
	γ-tocopherol	1	3.15	CPPCT042	11.8
		1	3.09	BPPCT020a	25.2
		4	3.50	UDP96-003	22.3
		4	3.27	PCHGMS55	14.0
	δ-tocopherol	1	2.25	CPPCT042	13.0
		4	4.87	PCHGMS55	26.6
		7	3.01	CPPCT033	22.8
**Fatty acids**	Oleic acid	2	3.52	UDP98-025	22.6
		7	3.64	CPPCT033	24.6
	Linoleic acid	2	2.30	UDP98-025	15.8
		7	3.14	CPPCT033	21.6
	Palmitic acid	3	4.00	BPPCT007	22.6
		7	3.62	CPPCT033	25.1
	Stearic acid	1	4.05	CPPCT042	25.3
		5	2.12	CPSCT022	11.0
		6	2.24	UDP98-412	19.4
		7	3.02	CPPCT033	21.2
	Palmitoleic acid	3	4.20	EPDCU3083	26.4
		5	3.12	CPSCT022	33.1
		7	3.19	CPPCT033	17.1
**Protein**	Total protein content	6	3.21	UDP98-412	17.0
		7	3.18	EPDCU3392	16.6
**Oil content**	Oil content	6	2.14	BPPCT008	12.0

### QTL for total protein and oil contents

Two QTL controlling the total protein content were detected in LG6 and LG7. The marker UDP98-412, located in the lowest part of the LG6 had an LOD of 3.21 and explained a phenotypic variance of 17 % (Table 2). The second QTL was found in the lowest part of the LG7 and had a similar effect, with an LOD of 3.18 explaining a phenotypic variance of 16.6 %. For the oil content, a QTL placed on chromosome 6 was observed close to the marker BPPCT008, with an LOD of 2.14 and explaining 12 % of the phenotypic variance.

### QTL for fatty acid composition

For fatty acid composition in almond, seven QTL were located in the first seven LG. The QTL for oleic acid concentration (C18:1) were mapped on LG2 (UDP98-025) and LG7 (CPPCT033), with an LOD of 3.52 and 3.64 respectively. Both QTL explained a total of phenotypic variance of 47.2. For linoleic acid (C18:2), the QTL were found in the same position as those detected for oleic acid. For palmitic acid (C16:0), again two QTL were detected, one in LG7 (CPPCT033) with an LOD 3.62, and another at the beginning of GL3 (BPPCT007) with an LOD of 4.0. These QTL explained a phenotypic variance of 47.7. For stearic acid (C18:0), four QTL were identified in LG1, LG5, LG6 and LG7, with the first three not yet identified for the other fatty acids. CPPCT042 in LG1 had an LOD of 4.05, CPSCT022 in LG5 an LOD of 2.12, UDP98-412 in LG6 an LOD of 2.24, and CPPCT033 in LG7 an LOD of 3.02. The phenotypic variance explained by these QTL was 25.3, 11.0, 19.4 and 21.2 % respectively, giving a total of 76.9 % of the total variation observed. For palmitoleic acid (16:1), three SSR loci (EPDCU3083, CPSCT022 and CPPCT033) were identified in the lower and middle region of GL3, GL5 and GL7. These markers, with LODs of 4.20, 3.12 and 3.19 respectively, explained most of the phenotypic variation (% Exp of 76.6 %).

It is significant that a QTL in LG2 affects the two main fatty acids, oleic and linoleic, and that another in LG 7 affects all five fatty acids studied, thus giving a genetic basis for the correlation between the different fatty acids inside the lipid pool (Table 3).

**Table 3:** **Average correlations between the chemical traits of the almond kernel (mean from different sources:**[12,13,22]**)**

**Trait**	**oil content**	**oleic acid**	**linoleic acid**	**palmitic acid**	**stearic acid**	**α-tocopherol**	**γ-tocopherol**	**δ-tocopherol**
protein content	−0.47**	−0.52**	0.08 ns	0.32**	0.09 ns	−0.16**	−0.67**	−0.59**
oil content	-	0.32*	−0.15**	−0.24**	0.15 ns	0.12 ns	0.35**	0.30**
oleic acid		-	−0.85**	−0.49**	0.20**	0.10 ns	0.48*	0.41**
linoleic acid			-	0.47**	0.31**	−0.23**	−0.27**	−0.23**
palmitic acid				-	−0.12 ns	−0.21**	−0.49**	−0.12*
stearic acid					-	−0.21**	−0.16**	−0.21*
α-tocopherol						-	0.52**	0.50**
γ-tocopherol							-	0.85**

ns, not significant; *P ≤ 0.05; **P ≤ 0.01.

### QTL for tocopherol

A total of five different QTL using the interval mapping approach were detected for the α-, γ-, and δ-tocopherol homologues (Table 2). Two QTLs for α-tocopherol were located in LG1 and LG4. In LG1, the marker CPPCT042 had an LOD of 3.21 and explaining a phenotypic variation of 20 %, whereas the second QTL was detected at the end of LG4, near the locus PCHGMS55, with an LOD of 2.26 and explaining 17.8 % the phenotypic variation. For γ-tocopherol, four QTL were detected. Two of them were in the same position as for α-tocopherol and two were newly identified also in the LG1 and LG4. The nearest locus newly found in LG1 was BPPCT020a, with an LOD of 3.09, whereas the nearest marker in LG4 was UDP96-003. The percentage of phenotypic variance explained by these QTL was 11.8 %, 25.2 %, 22.3 %, and 14 %, with a total of 73.3 %. For the third tocopherol homologue, δ, three QTL were located. Two of them coincided with those found for α and γ-tocopherol, although an additional QTL was located in LG7, between the locus UDP98-408 and CPPCT033, with an LOD of 3.01 and explaining a phenotypic variance of 22.8 %.

### Relations between QTL linked to chemical traits in almond

Pearson’s correlations between the chemical parameters controlled by the same QTL were observed in LG1, LG2, LG4 and LG7, but not on the other LGs where QTL were identified. These correlations at the mapping level correspond to the correlations already described for the chemical composition of the almond kernels (Table 3).

One QTL detected near the CPPCT042 marker in LG1 showed significant correlations for the traits controlling this locus, stearic acid and the three tocopherol homologues, agreeing with the negative correlation of stearic acid and the three tocopherol homologues (Table 3) and the positive correlation between the three tocopherol homologues.

Another QTL was detected near the PCHGMS55 marker in LG4, with the highest correlation between γ- and δ-tocopherol, and lower, but still significant correlation between α- and δ-tocopherol, and between α- and γ-tocopherol, as already established [22,42]. However, the correlation between α- and γ-tocopherol should have been negative since γ-tocopherol is a precursor in the synthesis of α-tocopherol [43]. This discrepancy may be due to the fact that the research on the pathways of tocopherol biosynthesis has been done in chloroplasts and not in seeds, where oil is really accumulated [44].

Significant correlations were found between traits controlling a QTL near the UDP98-025 marker in LG2. Oleic acid was negatively correlated with linoleic acid as expected (Table 3). This negative correlation may be explained by the fact that the pool of oleic acid appears to be controlled by its conversion to linoleic acid, probably as a result of the enzymatic activity of oleic desaturase [45]. Correlation coefficients greater than 0.71 or smaller than −0.71 have been suggested to be biologically meaningful [46], showing that this correlation is not influenced by climatic and environmental conditions and is genotype-dependent, as reflected by this QTL. Linoleic is a polyunsaturated fatty acid contributing significantly to the deterioration of food quality in the presence of oxidation catalysts such as enzymes, light and moisture [47]. So, if the concentration of linoleic acid decreases, food quality may increase.

Significant correlations were also found for a QTL positioned near the CPPCT033 marker in LG7. Negative correlations were found between δ-tocopherol and linoleic, palmitic and stearic acids and positive with oleic acid, as expected (Table 3). Negative and significant correlations were found between oleic, palmitic and stearic acids, and positive and low correlations were found between linoleic, palmitic and stearic acids.

### Breeding implications

Although a large number of QTL associated with different agronomic and economic traits have been identified in *Prunus* species using molecular markers, very few have been described in almond. The first trait was identified in almond using random amplification of polymorphic DNA (RAPD) markers [48], obtaining a very significant QTL for flowering time in LG4. The same trait was later confirmed in the same position [49] by using a candidate gene approach. Later nine QTL for traits such as blooming date, blooming density, productivity, leafing date, double kernels or ripening date, among others were mapped [50]. More recently new QTL associated with self-incompatibility have been identified [29]. However, no information is available on QTL linked to the chemical composition of the almond kernel, probably because of the high cost and labor of the chemical analyses [1]. However, this information would be essential in an almond breeding program taking into account the evolution of the market preferences towards natural products with nutritional and healthy properties.

As already pointed out in almond [12], the increase of the tocopherol content is a major goal included in the breeding programs of some species, such as rapeseed, oat, soybean and maize. In these species, genetics maps have been developed and mapping studies carried out with successful results in the last ten years, identifying in all cases QTL affecting α and γ-tocopherol [51-55]. Similarly, in this work two QTL were found associated at the same location with the three tocopherol homologues (α, γ and δ) in LG1 (CPPCT042) and LG4 (PCHGMS55).

A similar approach has been directed towards the fatty acid profile [19]. In some species, genetic studies have again been successfully carried out to detect QTL associated with the different fatty acids, such as in oil palm, coconut, maize, rapeseed and soybean [56-59], but almond has not yet received any attention from this point of view. This work has identified seven QTL located in all LGs, except in LG4 and LG8, related to the five major fatty acids of the almond kernel. The QTL identified in the LG7 (CPPCT033) was related to the five fatty acids included. In addition, the QTL located in LG2 (UDP98-025), has been found to be related to the two main fatty acids of almond, oleic and linoleic, whose concentrations are negatively correlated (Table 3). This QTL is not related to the three minor fatty acids, mapped by to other QTL.

Finally, three additional loci were mapped for protein content (LG6 and LG7) and for oil content (LG6). Both contents are interesting from a qualitative point of view, although negatively correlated (Table 3). This interdependence can be explained biochemically, since both fractions are formed during the ripening process from carbohydrates, abundant in the early stages of seed development but later decreasing throughout the ripening process [19].

In addition to their application in an almond breeding program, these QTL may be the first step in seeking candidate genes for the metabolic processes leading to component accumulation in the almond kernel. It has been already established that the Acetyl-CoA controls the synthesis of long-chain saturated fatty acids by integrating itself into the fatty acid synthase system. Several target genes have been suggested as controlling the production of fatty acids in plants. One Acetyl-CoA has been described and located in several species, such as soybean [60], sunflower [61], and *Camellia oleifera*[62]. Enough information is available on the *Prunus* genome, mainly the peach genome, to allow candidate genes to be proposed for some quality components. An Acetyl-CoA gene (Acetyl-CoA benzyl alcohol acetyltransferase) has been located on LG7 of the peach genome [24]. This gene could be a good candidate gene for lipid accumulation in almond since it is located within the interval where our QTL controlling the five fatty acids is positioned in LG7. Another gene called Enoyl-CoA hydratase from the isomerase family has been identified within the interval where our QTL controlling the two main fatty acids (oleic and linoleic) is positioned in LG2. Finally, two Acyl carrier protein (ACP) genes were located in the same regions as two QTL controlling stearic acid, in LG1 and LG6. Evidently this is only a first approach and further studies are needed in order to recognize more genes involved in the fatty acid biosynthetic pathway.

## Conclusions

The aim of any breeding program is to develop improved cultivars. The specific approach of any breeding program would depend on the clearly defined aims of this program, such as quality. Quality, however, is an extremely difficult aspect to define [63], and breeding for kernel quality is a demanding task in almond breeding. Some components are clear quality indices, such as high protein and oil content, as well as high oleic acid and tocopherol concentrations. However, all these traits are determined by a high number of interacting genes and regulatory factors. The knowledge of these genetic parameters would be very useful to make predictions of genetic progress in a breeding program. Although the genetic control of these traits has not yet been studied in almond, this new genetic information offers the opportunity for them to be considered in an almond breeding program for kernel quality. Increases in protein and oil contents may be sought simultaneously because even if the two components are negatively correlated, they are genetically independent.

In almond, fatty acid metabolism is controlled by a large number of diverse genes [64], in agreement with the QTL identified in this study, providing the first genetic framework for the chemical components of the almond kernel. The important number of QTL detected may improve the accuracy of the map and help validate these QTL as functional markers for marker-assisted breeding in almond.

## Methods

### Plant material and DNA isolation

The offspring studied included 77 individuals from the cross V × B obtained in the CITA almond breeding program of Zaragoza, Spain. The female parent ‘Vivot’ is a Spanish local cultivar, and the male parent ‘Blanquerna’ is a release from this program, obtained from ‘Genco’ × ‘AS-1’ pollination [65,66]. The study was located at 41°38’N and 0°53’W, at 220 m above sea level, at Zaragoza, Spain. These parents were selected because of their interesting characteristics, such as fruit quality and late blooming [67]. The trees are maintained as living plants in a nursery row using standard management practices. Approximately 50 mature fruits were randomly collected from each genotype. Fruits were cracked and seed coats removed by pouring in warm water (100 °C) during 5 min. Blanched kernels were dried until constant weight and ground in an electrical grinder to obtain fine flour [22]. The fruit was considered mature when the mesocarp was fully dry and split along the fruit suture and the peduncle was near to complete abscission [67].

The crops of two years were included for the analysis. The average values of the results of the two years were used because oil content and fatty acid and tocopherol concentrations have been found to show environmental stability [13,19]. The lack of the year effect was confirmed by the lack of significant differences between the values of the two years.

Genomic DNA was isolated from leaves following the CTAB extraction method based on Doyle and Doyle [68]. The DNA was quantified and diluted to 10 ng μL^-1^ to carry out PCR amplifications.

### Chemical analysis

Oil was extracted from 3 g of ground almond kernels in a Soxtec Avanti 2055 fat extractor (Foss Tecator, Höganäs, Sweden) [22]. Extracted oil was added to 10 μL of butylated hydroxytoluene methanolic solution as an antioxidant agent and kept in an amber vial at −20 °C in the freezer until required for analysis. The oil extraction was duplicated using 30 fruits of each genotype. The average values are reported as differences in weight of the dried kernel sample before and after extraction. The oil sample was used to prepare the methyl esters of the corresponding fatty acids (FAMEs) and for tocopherol content. The relative percentage of the different fatty acids in the oil was determined by capillary gas chromatography of FAMEs. These FAMEs were prepared by trans-etherification with KOH according to the official method UNE-EN ISO 5509:2000 [69]. The FAMEs were separated using a gas chromatograph HP 6890 and afterwards detected using a flame ionization detector, equipped with a capillary column (HP-Innowax 30 m x 0.25 mm i.d.) and 0.25 μm film thickness (Agilent Technologies, Waldbronn, Germany). The tocopherol content was determined according to a modification of a method already described [70]. The individual tocopherol isomers were analyzed using a reversed phase by high performance liquid chromatography, model 360 (Kontron, Eching, Germany) [12]. The protein fraction was determined through the total N content obtained by the Dumas method and applying a conversion factor as shown: % Protein = Kc * % total Nitrogen (Kc = 6.25). A sample of 0.2 g of almond flour was weighed and introduced into the analyser LECO FP-528 Protein/Nitrogen Analyzer (LECO Corporation, Saint Joseph, MI, USA).

### DNA marker genotyping, genetic mapping and QTL analysis

A total of 110 SSR markers previously described in other *Prunus* species (Table 1) were tested in the ‘V × B’ almond progeny to identify polymorphic markers between the two parents, pursuing a good coverage of the *Prunus* bin mapping T × E [59]. Those heterozygous in one or both parents and resulting in a good coverage of the T × E *Prunus* reference map were selected for analysis in the whole population. From the initial ‘V × B’ map [29], eight SSRs were additionally PCR amplified in order to increase the accuracy of the previous map, using the same conditions (Table 1). These eight SSRs were selected because they were designed for other *Prunus* species and showed a high level of polymorphism. Only four of these eight SSRs (CPPCT022, CPDCT027, BPPCT015 and CPPCT058) were polymorphic in both parents and, consequently, placed on the map. The other four SSRs (BPPCT012, BPPCT038, CPPCT043 and PMS67) did not show polymorphism in both parents and were not included in the map. The genetic map and segregation data used have been previously described [29]. Composite interval mapping was used for mapping QTL (MapQTL 4.0) [71]. When a QTL had a LOD score equal or higher than 2.0, it was declared significant.

## Competing interests

The authors declare that they do not have competing interests.

## Authors’ contributions

CFF performed the chemical analysis. AFM performed the SSR analysis and constructed the genetic map. AFM and CFF designed the study and carried out the QTL analysis. RSC obtained the plant material and supervised the study. AFM, CFF and RSC drafted the manuscript. All authors have read and approved the final manuscript.
